# Lifestyle Interventions for Hypertension and Dyslipidemia Among Women of Reproductive Age

**Published:** 2011-10-15

**Authors:** Cheryl L. Robbins, Patricia M. Dietz, Jennifer Bombard, Steven M. Schmidt, Michelle Tregear, Stephen J. Tregear

**Affiliations:** Centers for Disease Control and Prevention; Centers for Disease Control and Prevention, Atlanta, Georgia; Centers for Disease Control and Prevention, Atlanta, Georgia; Centers for Disease Control and Prevention, Atlanta, Georgia; Evidence-Based Decision and Policy Making Group, Manila Consulting Group, McLean, Virginia; Evidence-Based Decision and Policy Making Group, Manila Consulting Group, McLean, Virginia

## Abstract

**Introduction:**

Hypertension and dyslipidemia often precede cardiovascular disease. Lifestyle modifications help prevent these conditions, and referrals for women may be possible during reproductive health care visits. However, screening recommendations vary, which may affect screening rates. The objectives of this systematic review were to 1) assess the available literature on the effectiveness of lifestyle interventions, 2) review hypertension and dyslipidemia screening recommendations for consistency, and 3) report prevalence data for hypertension and dyslipidemia screening among women of reproductive age.

**Methods:**

We conducted a systematic literature search (January 1990-November 2010) for 1) randomized controlled trials on the impact of lifestyle interventions on cardiovascular disease risk factors in women of reproductive age, 2) evidence-based guidelines on hypertension and dyslipidemia screening, and 3) population-based prevalence studies on hypertension or dyslipidemia screening or both.

**Results:**

Twenty-one of 555 retrieved studies (4%) met our inclusion criteria. Lifestyle interventions improved lipid levels in 10 of 18 studies and blood pressure in 4 of 9 studies. Most guidelines recommended hypertension screening at least every 2 years and dyslipidemia screening every 5 years, but recommendations for who should receive dyslipidemia screening varied. One study indicated that 82% of women of reproductive age received hypertension screening during the preceding year. In another study, only 49% of women aged 20 to 45 years received recommended dyslipidemia screening.

**Conclusions:**

Lifestyle interventions may offer modest benefits for reducing blood pressure and lipids in this population. Inconsistency among recommendations for dyslipidemia screening may contribute to low screening rates. Future studies should clarify predictors of and barriers to cholesterol screening in this population.

## Introduction

Cardiovascular disease (CVD) is the leading cause of death in women (1) and the third leading cause of death among women of reproductive age (defined as 18-44 y unless otherwise specified) (2). The prevalence of hypertension and dyslipidemia, 2 major CVD risk factors, is relatively high among women of reproductive age. During 2005 through 2008, 8% of women aged 20 to 44 years had hypertension or were taking hypertension medication (2), and 11% had dyslipidemia (2). Although hypertension prevalence rates have remained stable during the last 10 years, approximately 40% of reproductive-aged adults (both men and women) with hypertension are unaware they have the condition (3).

Overall, women of reproductive age are not generally considered to be at high risk for CVD, but identification of hypertension and dyslipidemia has reproductive health significance. For women of reproductive age with hypertension, combined hormonal contraceptive methods are generally not recommended because they may increase CVD risk. Additionally, hypertension during pregnancy is associated with adverse outcomes such as preeclampsia, placenta abruption, preterm delivery, low birth weight, and infant death (4-7). Dyslipidemia is associated with polycystic ovary syndrome (8), the most common endocrine disorder among women of reproductive age and a leading cause of infertility (9). Dyslipidemia during pregnancy may also have adverse effects on both the fetus and mother (10,11).

Because women of reproductive age are at risk of becoming pregnant and drug therapy may pose risks to the fetus, lifestyle modifications are often the first line of treatment for hypertension or dyslipidemia. The effectiveness of lifestyle interventions such as exercise and diet on cardiovascular outcomes is well established for men and older women (12-18), but their effects on women of reproductive age are largely unknown. Reviewing hypertension and dyslipidemia screening recommendations for consistency between guidelines and understanding screening prevalence for women of reproductive age may clarify intervention referral opportunities. To our knowledge, no published reports have compared screening guidelines as they pertain to this population.

The primary objective of this systematic review was to evaluate the evidence from randomized controlled trials (RCTs) that have investigated the effects of lifestyle interventions on hypertension, dyslipidemia, or CVD illness and death in this population. Secondary objectives were to review hypertension and dyslipidemia recommendations for consistency and to report the prevalence of screening among women of reproductive age.

## Methods

### Data sources

Using electronic bibliographic databases (PubMed/MEDLINE, Cochrane Database of Systematic Reviews, and US National Guideline Clearinghouse), we conducted electronic searches on lifestyle interventions, national hypertension and dyslipidemia screening guidelines, and screening prevalence for women of reproductive age from January 1, 1990, through November 18, 2010. We also searched for relevant guidelines published by the American College of Obstetricians and Gynecologists. To conduct the search, we used a combination of free text terms and concepts derived from the National Library of Medicine's medical subject headings (Table 1). We applied additional filter options (English language and human studies) and related search features in iterative fashion to identify all relevant literature. In addition, we reviewed reference lists from retrieved articles and searched the grey literature, which consists of reports, studies, articles, and monographs produced by federal and local government agencies, private organizations, and educational institutions.

### Study selection

**Lifestyle interventions**

Two researchers (S.T., M.T.) searched the literature independently and selected studies on the basis of a priori inclusion criteria. We used researcher agreement to reconcile questions that arose about eligibility. We found no systematic reviews or meta-analyses of the effect of lifestyle interventions on CVD illness or death that focused on women of reproductive age or that parsed data to allow such analysis. Therefore, we used the following a priori inclusion criteria to identify individual studies: 1) RCTs or randomized crossover study designs; 2) enrolled 10 or more women of reproductive age or whose sample included subgroup analyses for women of reproductive age, or both; 3) full-length article; 4) outcomes of blood pressure, lipids, or CVD illness or death, or all; 5) diet or exercise intervention or both; and 6) published in the English language. Twenty-one of 555 studies (3.8%) met all a priori selection criteria and addressed 1 or more relevant outcomes (Figure).

**Figure 1.**
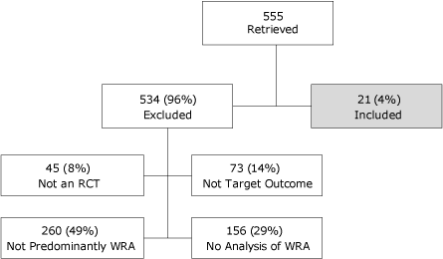
Selection of individual studies examining the effects of lifestyle interventions on hypertension, dyslipidemia, and cardiovascular disease illness and death among adult women of reproductive age. Abbreviations: RCT, randomized controlled trial; WRA, women of reproductive age.

**Screening recommendations**

Of particular interest to this review was an examination of guidelines likely to be in current use. As such, we focused on national-level US-based guidelines. We examined evidence-based guidelines produced under the auspices of medical specialty associations, relevant professional societies, and federal government agencies that had been reviewed, revised, or developed within the last 5 years (2005-2010), with the exception of 2 older seminal guidelines (the Joint National Committee on Prevention, Detection, Evaluation, and Treatment of High Blood Pressure [JNC 7] and the Adult Treatment Panel III cholesterol guidelines, both sponsored by the National Heart, Lung, and Blood Institute [NHLBI]), which continue to be referenced by other current guidelines. To be included in our assessment, a guideline had to meet the evidence-based criteria required for acceptance in the National Guideline Clearinghouse (www.guideline.gov/about/inclusion-criteria.aspx).

**Screening prevalence**

To describe current hypertension and dyslipidemia screening practices in the target population, we focused our searches on studies emanating from large population-based surveys in the United States, including the Behavioral Risk Factors Surveillance System (BRFSS), the Medical Expenditure Panel Survey (MEPS), the National Ambulatory Medical Care Survey (NAMCS), the National Health Interview Survey (NHIS), the National Health and Nutrition Examination Survey (NHANES), and the National Survey of Family Growth (NSFG).

**Data extraction**

We extracted data from included studies into comprehensive evidence tables to facilitate assessment of the quality of the individual studies. For the purposes of this report, we present details on the study setting and population, intervention, results (significant changes in outcomes in intervention groups relative to controls), and study quality from lifestyle intervention articles. We present included studies in descending chronological order, identified by first author and year (Tables 2, 3, and 4). We used a validated instrument designed to evaluate the internal validity of controlled studies to assess the quality of each of the studies. ECRI Institute (Plymouth Meeting, Pennsylvania) developed the instrument, which is available on request from the authors (19). We made no attempt to analyze or synthesize the findings quantitatively because of the large variation in the interventions assessed. Instead, we summarize the data qualitatively.

We extracted the following elements from hypertension and dyslipidemia guidelines: year, target population, recommended screening interval for all healthy and at-risk women of reproductive age, risk factors, and diagnostic criteria (Table 5). Lastly, we describe screening prevalence estimates for hypertension and dyslipidemia among women of reproductive age obtained from 2 studies that used population-based surveys.

## Results

### Lifestyle interventions

Of 555 retrieved references, we identified 21 studies that met our inclusion criteria, including diet interventions (n = 3), exercise interventions (n = 13), and combined diet and exercise interventions (n = 5). Eighteen studies examined the effect of an intervention on lipid levels, 9 examined blood pressure measures, and none focused on CVD illness or death. Study follow-up ranged from 6 weeks to 2 years. After reading the abstracts or the entire text, we excluded approximately 96% of the studies (534 of 555) largely because data precluded separate analyses of women of reproductive age (78%) (Figure). Additionally, 14% were excluded because our targeted outcomes were not addressed, and 8% were not RCTs or crossover study designs. We summarized findings from the trials that investigated the effect of diet, exercise, and combined diet and exercise interventions on systolic blood pressure (SBP), diastolic blood pressure (DBP), total cholesterol (TC), high-density lipoprotein cholesterol (HDL-C), low-density lipoprotein cholesterol (LDL-C), and triglycerides (TG).

**Diet alone**

We summarized data from 3 low- to moderate-quality, randomized crossover studies (20-22) involving 86 women of reproductive age that met our inclusion criteria (Table 2). All examined lipid levels and 1 also examined blood pressure measures (21). Study settings included Australia and the United States, and all studies compared low-fat to higher-fat diets and reported reduced mean values in TC (10.8-30.2 mg/dL), LDL-C (8.7-26.3 mg/dL), and HDL-C (3.4-10.1 mg/dL) for participants who followed low-fat diets. The study that examined blood pressure reported significant mean reductions in DBP (4.4 mm Hg) and arterial pressure (3.8 mm Hg) but not SBP for participants following a low-fat diet.

**Exercise alone**

Thirteen RCTs (12 moderate quality, 1 high quality) involved 482 women of reproductive age (Table 3). Exercise modes included resistance and endurance training, walking, running, and aerobics; study duration ranged from 6 to 40 weeks with varying intensities. Ten RCTs (23-32) examined lipids and 5 examined blood pressure (23,31,33-35). All lipid RCTs evaluated TC, LDL-C, HDL-C, and TG except 1, which did not examine LDL-C (32). Study settings were Brazil, Ireland, Nigeria, Turkey, and the United States.

Findings were mixed for the impact of exercise on lipid levels among women of reproductive age. In 3 of 10 trials, significant reductions in mean TC levels were found among those who received resistance training (12.8-16.3) or aerobics (28.2-39.8), compared with controls (25,27,29). Among 2 recent studies that examined TC, resistance training significantly reduced mean TC (12.8 mg/dL) compared with controls, but stair climbing did not (24,29). The other 7 studies that examined TC demonstrated no significant impact from exercise (23,24,26,28,30-32). Two of 9 trials examining LDL-C showed that stair climbing (24) and resistance training (29) led to significant mean improvements among women of reproductive age (6.6 and 13.9 mg/dL, respectively). However, 7 studies demonstrated no significant changes in LDL-C resulting from exercise (23,25-28,30,31). Only 1 study in 10 examining HDL-C demonstrated that exercise had a positive effect; it showed a mean increase of 6.5 mg/dL for step aerobics (25). Another study suggested undesirable effects of exercise on HDL-C (30), and 8 trials demonstrated no significant alterations to HDL-C (23,24,26-29,31-32). None of the RCTs demonstrated significant changes in TG in response to exercise.

Only 1 of the 5 trials examining blood pressure found an impact of exercise (35). That RCT showed continuous and interval aerobic training reduced SBP 10.8 to 12.4 mm Hg and DBP 2.5 to 2.6 mm Hg. None of the other RCTs demonstrated significant changes in blood pressure due to exercise.

**Combined diet and exercise interventions**

Five RCTs (1 low quality, 3 moderate quality, 1 high quality) representing 443 women of reproductive age (Table 4) examined TC, HDL-C, and TG; 3 examined LDL-C (36-38); and 3 examined blood pressure (38-40). Interventions varied in duration, ranging from 14 weeks to 2 years. Study settings were Canada, Finland, Italy, and the United States. The high-quality RCT was the most recent study, had the largest sample (120 obese women of reproductive age), the longest intervention period, and provided monthly sessions with a nutritionist and exercise trainer for the first year and bimonthly sessions in the second year (39). Interventions tested in the other RCTs included weight-reduction diets coupled with walking and group education (40) or with aerobics or resistance training (36); and aerobic exercise with low-fat diet and group education (38) or with fish diet (37).

Only 1 US RCT (38) found significant protective differences among the intervention groups relative to controls for TC and LDL-C reporting mean decreases in TC for the diet plus exercise group (10.8 mg/dL) and diet only (15.1 mg/dL), and in LDL-C for both intervention groups (11.2 and 10.9 mg/dL, respectively). One RCT focusing on obese women reported a 8.0 mg/dL mean increase in HDL-C among the intervention group (39). Four RCTs showed no effect of diet and exercise on HDL-C (36-38,40).

Three RCTs (37-39) found significant mean TG reductions (1.8-19.0 mg/dL) among intervention groups. The other 2 studies demonstrated no significant changes in TG (36,40).

Two studies (38,39) reported significant mean decreases in SBP (3.0-4.1 mm Hg) and DBP (2.0-3.0 mm Hg) among the intervention groups relative to controls. The other study that examined blood pressure demonstrated no significant changes as a result of diet and exercise (40).

### Current recommendations for hypertension and dyslipidemia screening and lifestyle modifications

Seven national US guidelines containing recommendations for hypertension and dyslipidemia screening were identified (Table 5). The guidelines for hypertension screening intervals and diagnostic criteria among women of reproductive age were generally consistent. Less agreement was observed between guidelines in the criteria for diagnosing dyslipidemia and cholesterol screening recommendations for women of reproductive age.

Five of the guidelines explicitly or by deferral to the NHLBI JNC7 guidelines (41) recommend hypertension screening every 2 years for adult women with optimal blood pressure (<120/80 mm Hg) and more frequently for those who have prehypertension or have other risk factors (41-46) (Table 5). Guidelines differ with regard to age at which hypertension screening should begin. The guidelines also vary in definitions of at-risk populations, but the following risk factors are consistent across guidelines: smoking, diabetes, obesity, physical inactivity, older age (>65 years for women), and having a personal or family history of premature CVD, hypertension, or dyslipidemia (<65 y for women and <55 y for men). The American Heart Association (AHA) also addresses pregnancy conditions and other gender-related comorbidities that identify women at risk (43). All organizations recommend that a series of standardized blood pressure measurements be taken over multiple visits before a diagnosis of hypertension is made, but there are nuanced differences between guidelines.

Lifestyle modifications, in particular exercise and weight reduction, were universally recommended by all guidelines as an integral part of CVD prevention and as first-line treatment for milder forms of hypertension. In addition, most recommend smoking cessation, maintaining a healthy diet rich in fruits and vegetables, and reduction of alcohol and sodium intake.

National cholesterol guidelines concur that women at increased risk of coronary heart disease (CHD) should be screened for dyslipidemia. However, only AHA (43) and NHLBI Adult Treatment Program III (48) recommend screening women of reproductive age (≥20 y) who are not at increased risk. Increased risk is generally defined by the presence of 1 or more of the following: diabetes, previous personal history of CHD or noncoronary atherosclerosis, a family history of premature CVD, current tobacco use, hypertension, or obesity. Screening frequency recommendations are similar, generally every 5 years, with shorter intervals for women whose lipid levels are close to warranting therapy, and longer intervals for those not at increased risk if they have repeatedly had normal lipid levels. Most organizations recommend that a full lipid profile be obtained and that lipid screening be performed after a fast of 9 to 12 hours. However, there is some disagreement between guidelines about the need for fasting blood levels and the value of including triglycerides as a part of the initial tests (49). Furthermore, consensus about diagnostic criteria for dyslipidemia is lacking (Table 5). Most guidelines recommend the same CVD risk reduction lifestyle modifications for cholesterol management as mentioned previously for hypertension, except sodium reduction. Additionally, they emphasize high-fiber, low-fat diets.

**Screening prevalence**

We identified only 2 hypertension and dyslipidemia screening prevalence studies that used population-based data and included women of reproductive age. The sole report that examined hypertension screening rates among women of reproductive age (defined as 14-44 y) was based on 1988 NSFG data (50) and estimated annual hypertension screening within the preceding year to be 82.3%. Predictors of hypertension screening among women of reproductive age in that report included having had a family planning visit in the previous 12 months, current or recent pregnancy, history of hypertension, older age, black race, and higher education or income (50). Only 1 study reported cholesterol screening rates among women of reproductive age, and it used 1999-2006 National Health and Nutrition Examination Survey (NHANES) data (51). That study reported 49% of women aged 20 to 45 years with no CHD risk factors received cholesterol screening within the preceding 5 years. Screening rates were 52% among women with 1 risk factor and 69% among those with CHD or CHD equivalent risk.

## Discussion

Limited conclusions can be drawn about lifestyle interventions in women of reproductive age because of the small number of included RCTs, the heterogeneity of interventions examined, and the lack of consistent findings across studies. Lifestyle interventions improved dyslipidemia in 10 of 18 studies and hypertension in 4 of 9 studies. Stronger benefit was seen on levels of TC and LDL-C than on HDL-C or TG. Improvements in systolic blood pressure were seen in 3 of 9 studies that examined blood pressure changes. Diastolic blood pressure improved in 4 of 9 studies. Follow-up tended to be short-term (1-2 y), and most samples comprised healthy women of reproductive age.

Our assessment of the effectiveness of lifestyle intervention is consistent with reviews conducted on low-risk populations. A systematic review of lifestyle interventions among healthy adult men and women also concluded that lifestyle interventions offered marginal short-term benefit on blood pressure and, to a lesser degree, lipids (52). Two meta-analyses examined the effect of aerobic exercise on blood pressure and found modest reductions among normotensive, mostly older women (13,53). The effects of lifestyle interventions on lipids appear to be strongest for LDL-C and TC and weaker for any benefit to HDL-C or TG, consistent with similar investigations (14,54). Previous reports indicated mixed findings regarding the effect of exercise on TG and HDL-C levels; improvements were reported for physically inactive subjects primarily. Indeed, that was the case for the studies in which we found improvements in TG and HDL-C (38,39). Finally, exercise duration may be the most important predictor of change to HDL-C (55,56); the 2 studies reporting improvements in TG and HDL-C levels had the longest intervention duration (1-2 y).

The review of guidelines revealed that diagnostic criteria and screening recommendations for dyslipidemia vary. Optimal screening tests include measurement of total and HDL-C levels or apolipoproteins without fasting and without regard to triglycerides (49). Updated NHLBI guidelines for hypertension and dyslipidemia screening are anticipated in 2012.

We found only 1 study that examined prevalence of hypertension screening and another for dyslipidemia screening among women of reproductive age. One study reported 82% of women of reproductive age received hypertension screening within the preceding year (50), which is higher than a current estimate of 75% screened (according to unpublished National Health Interview Survey [NHIS] data analyses, women aged 14-44 y, 2008). However, nearly 90% of women of reproductive age get hypertension screening within the recommended interval of every 2 years (NHIS data analyses, 2008). Kuklina et al also reported that 49% to 69% of women aged 20 to 45 years had their cholesterol checked in the previous 5 years (51), which is consistent with estimates from 2008 NHIS unpublished analyses for the same population (64%). The lack of consensus among dyslipidemia screening guidelines may be the reason for lower screening rates in this population.

Few studies provide detailed examination of hypertension and dyslipidemia screening prevalence among women of reproductive age. Perhaps this gap in the literature exists because young people tend to be healthy and the age gradient is marked in these conditions, so women of reproductive age have not been considered a target for screening surveillance. However, identification of high-risk subpopulations and clarification of screening recommendations may prevent the onset of hypertension, dyslipidemia, and other chronic conditions such as diabetes among those at increased risk for CVD.

Substantial body of evidence establishes that diet and exercise improve hypertension and dyslipidemia, but that literature is predominantly based on studies of men and older women. Individual study samples included in this review may lack the power to detect the benefits of lifestyle interventions among healthy populations. For example, pooled results among RCTs that enrolled healthy older women detected significant effects between aerobic exercise and blood pressure, although the individual RCT findings were not significant (53). Pooling studies in meta-analyses can add the needed statistical power to detect modest short-term benefits of lifestyle interventions, but not enough studies are focused on women of reproductive age to do this.

Women of reproductive age are a population in need of CVD screening and early intervention. Lifestyle modifications are appropriate initial therapies for most patients and may reduce CVD risk through mechanisms other than lowering LDL-C or blood pressure, such as through smoking cessation, weight reduction, and increased physical activity (48). Moreover, a dose-response effect of physical activity on CHD risk suggests that higher intensity exercise conveys greater benefit (57-59).

To our knowledge, this is the first published systematic review of RCTs examining the effects of lifestyle interventions on hypertension, dyslipidemia, or CVD among women of reproductive age. Its strengths include a review of the grey literature, report of study flow, and assessment of the quality of included RCTs. The geographic breadth of included RCTs spanned Africa, Asia, Europe, Oceania, and North and South America. Studies from Europe and North America were most prevalent; thus, results are generally representative of women of reproductive age from those regions. However, racial composition was addressed in only one-third of the studies (20,22,28,29,31,33,34), and only 5 included minority women (20,22,31,33,35). Given racial differences in hypertension and dyslipidemia screening (50,60,61) and the need to explore lifestyle interventions' benefits in high-risk subpopulations of women of reproductive age, future RCTs should recruit sufficient numbers of at-risk women of reproductive age, including African American and obese women. The main limitation of this review is the possibility of missed studies. We did not search non–English-language literature, and it is possible that RCTs have been published in other languages. We also may have missed potentially relevant studies that are not indexed in PubMed.

Given the reproductive health importance of identifying hypertension or dyslipidemia among women of reproductive age, surveillance of hypertension and dyslipidemia screening in this population is needed. Lifestyle interventions may offer modest short-term benefits for reducing blood pressure or lipids among healthy women of reproductive age that may lead to larger long-term benefits. Further research is needed to clarify predictors of and barriers to cholesterol screening in this population and to investigate the long-term benefits of lifestyle interventions for women of reproductive age.

## Figures and Tables

**Table 1:** Medical Subject Headings and Free-Text Search Terms Used in Electronic Searches

**Terms**	Medical Subject Headings	Free-Text
**Disease-specific–related terms**	Hypertension Hypertension/epidemiology/*prevention and control Dyslipidemia* Dyslipidemia*/epidemiology/*prevention and control Hyperlipidemia* Hyperlipidemia*/epidemiology/*prevention and control Hypercholesterolemia Cholesterol, LDL Cholesterol, HDL Cardiovascular disease* Cardiovascular disease*/epidemiology/*prevention and control Complications	Blood pressure, high Hyperlipemia Hyperlipidemia Lipemia Lipidemia Lipid disorders Cholesterol Low-density lipoprotein cholesterol High-density lipoprotein cholesterol Triglycerides Adverse events Adverse effects
**Screening and treatment-related terms**	Mass screening Exercise Diet Utilization Therapy	Screening Screening trends Exercise Physical activity Diet Lipid analysis Treatment Disease management Disease prevention
**Other**	Cross-sectional survey Health surveys Review Meta-analysis Guideline	NHANES NHIS BRFSS MEPS NAMCS Population survey Systematic review Meta-analysis Clinical practice guideline Evidence-based guidelines Standards

Abbreviations: LDL, low-density lipoprotein; HDL, high-density lipoprotein; NHANES, National Health and Nutrition Examination Survey; NHIS, National Health Interview Survey; BRFSS, Behavioral Risk Factor Surveillance System; MEPS, Medical Expenditure Panel Survey; NAMCS, National Ambulatory Medical Care Survey. Asterisk (*) indicates wildcard in search.

**Table 2:** Selected Characteristics of Randomized Controlled Trials Examining Cardiovascular Effects of Diet

**Study, Setting, and Quality^a^**	Study Population^b^	Intervention	Results
Gerhard et al 2000 (20) Academic: Oregon Health Sciences University Portland, Oregon, United States Moderate	22 healthy white and African American premenopausal women aged 18-45 y living in the Portland area who participated in a previous study	Randomized crossover design assignment to diet order Intervention — Low- to high-fat/cholesterol diet and high- to low-fat/cholesterol diet Protocol — Follow randomly assigned order of diets. Start first diet x 4 wks; 4-wk washout period; follow other diet for 4 wks	Low-fat and cholesterol diets were associated with decreased TC decreased HDL-C decreased LDL-C increased TG White women (n = 9) had higher VLDL-C cholesterol concentrations and TG than African American women (n = 13). Otherwise, no racial differences were noted. Small sample sizes may have impaired ability to detect differences.
Pellizzer et al 1999 (21) Hospital: Austin Hospital Victoria, Australia Low	25 healthy, nonsmoking, premenopausal women aged 18-45 y in 20% of ideal body weight	Randomized, crossover design assignment to 1 of 2 diets Intervention — 1st: Low in total and saturated fat (25%) and cholesterol; 2nd: High in total and saturated fat (40%) and cholesterol Protocol — Follow randomly assigned order of diets. Start first diet, follow for 2 wks; 2-wk washout; Follow other diet for 2 wks	Low-fat diets associated with decreased DBP decreased TC decreased HDL-C decreased LDL-C no significant change in SBP weight did not change significantly
Ginsberg et al 1998 (22) Multicenter trials: Columbia University, Pennington Biomedical Research Center, Pennsylvania State University, University of Minnesota, United StatesModerate	39 healthy, normolipidemic, premenopausal women recruited from 4 research centers; mean age, 31 y	Randomized, crossover design Intervention — Diet A: Average American diet with 37% fat, including 16% SFA; diet B: Step 1 diet with 30% fat including 9% SFA; diet C: Low-SFA diet with 26% fat including 5% SFA Protocol — Randomly assigned a diet sequence that includes each diet: ABC, ACB, BAC, BCA, CAB, or CBA Assigned diets were followed for 8 wks, followed by 4- to 6-wk washout between diets, and next diet	Relative to average American diet, Step 1 and Low-SFA diets associated with decreased TC decreased HDL-C decreased LDL-C no significant change in TG

Abbreviations: TC, total cholesterol; HDL-C, high-density lipoprotein cholesterol; LDL-C, low-density lipoprotein cholesterol; TG, triglycerides; VLDL-C, very low-density lipoprotein cholesterol; DBP, diastolic blood pressure; SBP, systolic blood pressure; SFA, saturated fatty acids.

a Quality was defined as ratings based on ECRI Institute 25-item validated instrument (19).

b Number of subjects limited to those who completed the study.

**Table 3:** Selected Characteristics of Randomized Controlled Trials Examining Cardiovascular Effects of Exercise

**Study, Setting, and Quality^a^**	Study Population^b^	Intervention	Results
Ciolac et al 2010 (23) Brazil Moderate	44 healthy female college students Mean age by group: Aerobic interval training = 24.4 y; continuous exercise training = 26.6 y; control = 25.3 y Intervention, n = 16; control, n = 12	Intervention. Five min warm up, 15 min of calisthenics, and either aerobic interval training (AIT, n = 16) or continuous exercise training (CET, n = 16) for 40 min for 3 times/wk for 16 weeks Intensity. AIT = 50%-90% max ventilation oxygen uptake (VO_2MAX_) CET = 60%-70% VO_2MAX_	Relative to controls, interventions associated with no significant change in TC no significant change in LDL-C no significant change in HDL-C no significant change in TG no significant change in SBP no significant change in DBP
Boreham et al 2005 (24) Northern Ireland, UK High	15 sedentary, but otherwise healthy, young female college students Mean age, 18.8 y Intervention, n = 8; control, n = 7	Intervention. Stair-climbing program 5 times/wk for 8 wks Intensity. Progressive starting with 2 sets (199 stairs) at 90 steps/min and working up to 5 sets	Relative to controls, interventions associated with: decreased LDL-C no significant change in TC no significant change in HDL-C no significant change in TG
Kin Isler et al 2001 (25) Ankara, Turkey Moderate	45 sedentary female college student volunteers Mean age by group: Step aerobics = 21.9 y; aerobic dancing = 20.2 y; control = 21.9 y Intervention, n = 30; control, n = 15	Intervention. Step aerobics (n = 15) or aerobic dancing (n = 15) for 45 min, 3 times/wk for 8 wksIntensity. Sixty to 70% heart rate reserve	Relative to controls, both interventions associated with decreased TC no significant change in TG no significant change in LDL-C Relative to controls, step aerobics associated with increased HDL-C
LeMura et al 2000 (26) Pennsylvania, United States Moderate	45 college-aged, nonsmoking female students with no regular physical activity for 4 mo before study, and taking no medications known to alter lipid metabolism Mean age = 20.4 y Intervention, n = 33; control, n = 12	Intervention. Resistance training (n = 11), aerobic training (n = 10), or cross training (n = 12) for 3 times/wk for 16 wks Intensity. Resistance = Nautilus 3 times per wk; aerobic = 3 times per wk; cross-training = aerobics 2 times/wk and Nautilus 2 times/wk Control. No training during 16 wks.	Relative to controls, interventions associated with no significant change in TC no significant change in LDL-C no significant change in HDL-C no significant change in TG
Prabhakaran et al 1999 (27) Virginia, United States Moderate	24 sedentary, premenopausal healthy women recruited by campus newspaper and word of mouth Mean age by group: resistance training, 28.0 y; control, 26.0 y Intervention, n = 12; control, n = 12	Intervention. Supervised, intensive, resistance exercise training sessions 45-50 min/d, 3 d/wk for 14 wks Control. Nonexercising	Relative to controls, intervention associated with decreased TC no significant change in LDL-C no significant change in HDL-C no significant change in TG no significant change in body mass
Duey et al 1998 (33) Alabama, United States Moderate	25 sedentary African American women Mean age by group: intervention, 23.6 y; control, 22.2 y Intervention, n = 16; control, n = 9	Intervention. Endurance exercise training sessions 20 min/d (plus warm-up and cool-down), 3 d/week for 6 wks Intensity. Weeks 1-2: 60% peak oxygen uptake (VO_2peak_); weeks 3-4: 65% VO_2peak_; weeks 5-6: 70% VO_2peak_ Control. Usual diet and physical activity	Relative to controls, intervention associated with no significant change in SBP no significant change in DBP
Santiago et al 1995 (28) Minnesota, United States Moderate	27 mostly white, healthy female volunteers aged 22-40 y, nonsmokers, not pregnant, sedentary, body mass index <31 kg/m^2^ Intervention, n = 16; control, n = 11	Intervention. Brisk treadmill walking for 3 miles, 4 d/wk for 40 wks Intensity. 72% maximal heart rate Control. Sedentary	Relative to controls, intervention associated with no significant change in HDL-C no significant change in LDL-C no significant change in TC no significant change in TG no significant change in body composition
Boyden et al 1993 (29) Arizona, United States Moderate	88 white, healthy female volunteers aged 28-39 y, smoked ≤10 cigarettes/d, inactive, not overweight or obese Intervention, n = 46; control, n= 42	Intervention. Resistance exercising for 1 hour, 3 d/wk for 5 mos Intensity. Load major muscle groups in the arms, legs, trunk, and lower back Control. Inactive	Relative to controls, intervention associated with decreased LDL-C decreased TC no significant change in HDL-C no significant change in TG
Hinkleman et al 1993 (30) California, United States Moderate	36 premenopausal female volunteers aged 25-45 y, not presently exercising or dieting, 10%-40% overweight, nonsmokers, no history of alcohol or drug abuse Intervention, n = 18; control, n = 18	Intervention. Walking 45 min, 5 d/wk for 15 wks Intensity. Sixty percent heart rate Control. Non-exercising	Relative to controls, intervention associated with no significant change in LDL-C no significant change in TC no significant change in TG decreased HDL-C significant change in body weight no significant change in body fat
Katz et al 1992 (34) Ohio, United States Moderate	21 white, healthy female volunteers aged 18-28 y, nonsmokers, inactive, no history of cardiovascular disease Intervention, n = 13; control, n= 8	Intervention. Low-intensity resistance exercise training on Nautilus 30 min/d, 3 d/wk for 6 wks Control. Not trained	Relative to controls, intervention associated with no significant change in SBP no significant change in DBP
Duncan et al 1991 (31) Texas, United States Moderate	53 mixed-race, healthy women aged 20-40 y, nonsmokers, sedentary, "light or nondrinkers" Intervention, n = 43; control, n = 10	Intervention. Aerobic walking (n = 13), brisk walking (n = 12), or strolling (n = 18) 4.8 km, 5 d/wk for 24 wks Intensity. Aerobic walkers, 8.0 km/h; brisk walkers, 6.4 km/h; strollers, 4.8 km/h Control. Sedentary	Relative to controls, intervention associated with no significant change in seated blood pressure no significant change in TC no significant change in LDL-C no significant change in HDL-C no significant change in TG
Edin et al 1990 (32) Minnesota, United States Moderate	17 healthy, nonpregnant women aged 18-40 y, sedentary, nonsmokers with body weight within 80%-120% of standard body weight for height range Intervention, n = 10; control, n = 7	Intervention. Aerobic exercise on trampoline 30 min, 5 d/wk for 11 wks Intensity. Training heart rate zone of 70%-85% of maximal heart rate Control. Sedentary	Relative to controls, intervention associated with no significant change in TC no significant change in HDL-C no significant change in TG
Oluseye et al 1990 (35) Ibadan, Nigeria Moderate	42 sedentary Nigerian women, aged 20-50 yIntervention, n = 30; control, n = 12	Intervention. Interval Aerobic Training Protocol (ITP) (n = 15) or Continuous Aerobic Training Protocol (CTP) (n = 15) 50 min, 3 d/wk for 12 wks Intensity. Progressive 65%-95% of maximal heart rate with increases of 5% every 2 wks Control. Sedentary	Relative to controls, interventions associated with decreased SBP decreased DBP

Abbreviations: TC, total cholesterol; LDL-C, low-density lipoprotein cholesterol; HDL-C, high-density lipoprotein cholesterol; TG, triglycerides; SBP, systolic blood pressure; DBP, diastolic blood pressure.

a Quality ratings based on ECRI Institute 25-item validated instrument (20).

b Number of subjects limited to those who completed the study.

**Table 4:** Selected Characteristics of Randomized Controlled Trials Examining Cardiovascular Effects of Diet and Exercise

**Study, Setting, and Quality^a^**	Study Population^b^	Intervention	Results
Esposito et al 2003 (39) Naples, Italy High	120 premenopausal, sedentary, obese, nonpregnant women aged 20-46 y recruited from the outpatient department for weight loss of the teaching hospital. Exclusion criteria: dieting within previous 6 mos, type 2 diabetes or impaired glucose tolerance, hypertension, cardiovascular disease, psychological problems, alcohol abuse, smokers, and any medication use Intervention, n = 60; control, n = 60	Intervention. Individual counseling on increasing physical activity for 2 y; small group sessions on reducing dietary calories, personal goal setting, and self-monitoring Intensity. Monthly sessions with a nutritionist and exercise trainer for 12 mos, bimonthly for 12 mos Control. Monthly group education sessions	Relative to controls, intervention associated with decreased SBP decreased DBP decreased TG increased HDL-C no significant change in TC Blood pressure and cholesterol were secondary outcomes of the study; primary outcomes were inflammatory markers. No adjustments were made for multiple comparisons.
Janssen et al 2002 (36) Ontario, Canada Moderate	38 premenopausal, upper-body obese, women with stable weight in 6 mos before study, taking no medications, with regular menses Mean age by group: diet and aerobics = 37.5 y; diet and resistance = 34.8 y; diet only = 40.1 y Intervention, n = 25; control, n = 13	Intervention. Weight maintenance diet for 2 wks before pretreatment testing Diet and aerobics (DA) (n = 11); diet and resistance (DR) (n = 14); weight reduction diet for 16 wks Intensity. Diet = 1000 kcal deficit diet; DA = 15-60 min sessions of aerobic exercise for 5 d/wk; DR = 5-10 min cycling and 30 min sessions of resistance exercise 3 d/wk Control. Diet only	Relative to controls, intervention associated with no significant change in TC no significant change in LDL-C no significant change in HDL-C no significant change in TG
Fogelholm et al 2000 (40) UKK Institute, Tampere, Finland Moderate	74 premenopausal, healthy, sedentary female volunteers aged 30-45 y with body mass index 30-45 kg/m^2^ and stable weight over previous 3 months, nonbingeing, not taking medication other than birth control, and not pregnant, lactating, or smoker Intervention, n = 47; control, n = 27	Intervention. Twelve wks weight reduction diet followed by maintenance program for 40 wks with weekly small group meetings and random assignment to walk-1 (n = 24), walk-2 (n = 23), or control (n = 27); unsupervised 2-year follow-up Intensity. Walk-1 average 2-3 h weekly; walk-2 group average 4-6 h weekly Control. Diet counseling with no change in exercise during maintenance program	Relative to controls, interventions associated with no significant change in TC no significant change in HDL-C no significant change in TG no significant change in SBP no significant change in DBP Blood pressure and cholesterol were secondary outcomes of the study; primary outcomes were body weight, fat mass, and waist circumference.
Ågren et al 1991 (37) University setting in Finland Low	99 healthy female students (age not specified) Intervention, n = 76; control, n = 23	Intervention. Fish diet (n = 22), exercise (n = 27), or fish diet and exercise (n = 27) for 14 wks Intensity. Fish diet: offered meal containing 150g fish for 5 d/wk but uptake was 3.5 d/wk; aerobic exercise: advised to obtain 30 min moderate intensity aerobic activity ≥3 times/wk but uptake was 1.3 times/wk Fish diet and aerobic exercise: as described above	Relative to controls, fish diet and exercise interventions associated with decreased TG no significant change in TC no significant change in LDL-C no significant change in HDL-C
Wood et al 1991 (38) Stanford University, Palo Alto, California, USA Moderate	112 healthy, sedentary, moderately overweight, nonsmoking, female volunteers aged 25-49 y, consuming <4 alcoholic drinks/d, not taking medication that could affect blood pressure or cholesterol, not lactating, pregnant, or taking oral contraceptives in past 6 mos, and not planning pregnancy in next 2 years Intervention, n = 73; control, n = 39	Intervention. Diet-only (n = 31) or diet and exercise (n = 42) Intensity. Daily diet consisting of 55% carbohydrates and 30% fat with ≤10% saturated fat and ≤300 mg cholesterol; weekly group sessions for 3 mos followed by every other week for 3 mos and monthly for 6 mos; supervised progressive aerobic exercise: brisk walking and jogging 3 d/wk at 25 min per session increasing to 45 min per sessions by 4th month Control. Asked to maintain usual diet and exercise habits	Relative to controls interventions associated with decreased TC decreased LDL-C decreased TG decreased SBP decreased DBP no significant change in HDL-C Relative to diet only group, diet and exercise associated with increased HDL-C

Abbreviations: SBP, systolic blood pressure; DBP, diastolic blood pressure; TG, triglycerides; HDL-C, high-density lipoprotein cholesterol; TC, total cholesterol; LDL-C, low-density lipoprotein cholesterol.

a Quality ratings based on ECRI Institute 25-item validated instrument (20).

b Number of subjects limited to those who completed the study.

**Table 5:** National Blood Pressure and Cholesterol Screening Guidelines for Diagnosing Hypertension and Dyslipidemia in Women

**Developer and Year**	Who and When to Screen	Risk Factors	Diagnostic Criteria
**Hypertension**			
American Academy of Family Physicians (AAFP) (42) 2011	WHO: Women aged ≥18 y References US Preventive Services Task Force (USPSTF) WHEN: Healthy and at-risk adults: not stated but refers to JNC7	References USPSTF Smoking, diabetes, abnormal blood lipid values, older age, sex, sedentary lifestyle, and obesity	References USPSTF SBP ≥140 mm Hg and/or DBP ≥90 mm Hg ≥2 elevated readings obtained on ≥2 visits over a period of 1 to several weeks
American College of Obstetricians and Gynecologists (ACOG) (62) 2007	WHO: Women aged ≥18 y WHEN: Healthy and at-risk adults: not stated	African American, older age, prehypertension, family history of hypertension, lifestyle factors associated with hypertension	See criteria used by the National Heart, Lung, and Blood Institute (NHLBI) Joint National Committee on Prevention, Detection, Evaluation, and Treatment of High Blood Pressure (JNC7)
American Heart Association (AHA) (43) 2011	WHO: Women aged ≥20 y WHEN: Healthy and at-risk adults: not stated, but refers to JNC7	High risk: CHD, cerebrovascular disease, PAD, abdominal aortic aneurysm, end-stage or chronic renal disease, diabetes mellitus, 10-y Framingham global risk >20% At risk: cigarette smoking, prehypertension, dyslipidemia, obesity, poor diet, physical inactivity, obesity, family history of premature CVD, metabolic syndrome, hypertension, dyslipidemia, evidence of vascular disease, subclinical atherosclerosis, metabolic syndrome, poor exercise capacity, systemic autoimmune collagen-vascular disease, history of preeclampsia, gestational diabetes, or pregnancy-induced hypertension	SBP ≥140 mm Hg or DBP ≥90 mm Hg, or SBP ≥130 mm Hg or DBP ≥80 mm Hg if chronic kidney disease or diabetes is present
Institute for Clinical Systems Improvement (ICSI) (44,45) 2008, 2009	WHO: Average-risk, asymptomatic women aged ≥18 y WHEN: Healthy adults: every 2 years At-risk adults: prehypertension, 1 y; stage 1 hypertension, 2 mos; stage 2 hypertension, within 1 mo	Hypertension, age, diabetes mellitus, elevated LDL-C, low HDL-C, estimated GFR <60 mL/min, microalbuminuria, family history of premature CVD, obesity, physical inactivity, tobacco use, target organ damage to heart, brain, chronic kidney disease, PAD, or retinopathy	Prehypertension: SBP = 120-139 mm Hg or DBP = 80-89 mm Hg Stage 1 hypertension: SBP ≥140-159 mm Hg or, DBP ≥90-99 mm Hg Stage 2 hypertension: SBP ≥160 mm Hg or, DBP ≥100 mm Hg Initial visit plus 2 follow-up visits, each including 2 measures per visit
NHLBI JNC7 (41) 2003	WHO: Adult women WHEN: Healthy adults: every 2 y At-risk adults: prehypertension, 1 y; stage 1 hypertension, 2 mos; stage 2 hypertension, within 1 mo, or if SBP ≥180 mm Hg or DBP ≥110 mm Hg, treat immediately or within 1 wk depending on clinical situation and complications	Hypertension, older age, diabetes mellitus, elevated LDL-C or total cholesterol or low HDL-C, estimated GFR <60 mL/min, family history of premature CVD, microalbuminuria, obesity, physical inactivity, tobacco usage, target organ damage to heart, brain, chronic kidney disease, PAD, or retinopathy	Prehypertension:SBP = 120-139 mm Hg or DBP = 80-89 mm Hg Stage 1 hypertension, SBP ≥140 mm Hg or DBP ≥90 mm Hg Stage 2 hypertension, SBP ≥160 mm Hg or DBP ≥100 mm Hg Average of ≥2 seated blood pressure measurements per visit on ≥2 office visits
US Preventive Services Task Force (USPSTF) (46) 2007	WHO: Women aged ≥18 y without known hypertension WHEN: Healthy and at-risk adults: not stated but refers to JNC7	Smoking, diabetes, abnormal blood lipid values, age, sex, sedentary lifestyle, and obesity	Initial visit ≥2 follow-up visits within a few weeks to 1 mo, each including 2 measures per visit
Veterans Health Administration (VHA) (47) 2004, revised 2005	WHO: Women aged ≥17 y WHEN: Healthy adults: annually At-risk adults: stage 1 hypertension, 2 mos; stage 2 hypertension, within 1 mo	Tobacco use, dyslipidemia, diabetes mellitus, obesity, physical inactivity, microalbuminuria or estimated GFR <60 mL/min, age (>65 y for women), family history of CVD for women younger than 65 or men younger than 55	Stage 1 hypertension, SBP ≥140 mm Hg or DBP ≥90 mm Hg Stage 2 hypertension, SBP ≥160 mm Hg or DBP ≥100 mm Hg 2 separate visits within 1-2 mo or sooner, each including ≥2 measures per visit
**Dyslipidemia**			
AAFP (42) 2011	WHO: At-risk women aged 20-45 yReferences USPSTF. WHEN: Healthy adults: No recommendation At-risk adults: Uncertain; reasonable option is at least once every 5 years, shorter intervals for people who have lipid levels close to those warranting therapy, longer intervals for those with repeatedly normal lipid levels	See USPSTF	See USPSTF
ACOG (62) 2007	WHO: Women aged ≥45 y and younger women with risk factorsWHEN: Healthy and at-risk adults: not stated but refers to Adult Treatment Panel III (ATP III)	Presence of CHD, diabetes, other clinical forms of atherosclerotic disease, cigarette smoking, hypertension, low HDL-C, family history of premature CHD, and older age	Recommends fasting and no exercise, tobacco use, or caffeine before measurementRefers to ATP III
AHA (43) 2011	WHO: Women aged ≥20 y WHEN: Healthy and at-risk adults: no recommendation	High risk: CHD, CVD, PAD, abdominal aortic aneurysm, end-stage or chronic renal disease, diabetes mellitus, 10-y Framingham global risk of ≥10% At risk: cigarette smoking, prehypertension, dyslipidemia, obesity, poor diet, physical inactivity, family history of premature CVD, metabolic syndrome, evidence of subclinical atherosclerosis, poor exercise capacity, systemic autoimmune collagen-vascular disease, history of preeclampsia, gestational diabetes, or pregnancy-induced hypertension	LDL-C ≥100 mg/dL HDL-C ≤50 mg/dL TG ≥150 mg/dL Non-HDL-C ≥130 mg/dL
ICSI (45,63) 2009	WHO: Women aged ≥45 y and at-risk women aged 20-44 y WHEN: Healthy adults: every 5 yAt-risk adults: every 3-12 mos	First-degree relatives with total cholesterol >300 mg/dL or history of premature CHD; personal history of CHD, CVD, peripheral vascular disease, diabetes mellitus, metabolic syndrome, current dyslipidemia Also refers to ATP III definitions of high risk	TC ≥200 mg/dL LDL-C ≥130 mg/dL TG ≥200 mg/dL HDL-C <40 mg/dL
NHLBI, National Cholesterol Education Program, ATP III (48) 2002	WHO: Women aged ≥20 y WHEN: Healthy adults: at least once every 5 y At-risk adults: more frequent measurements are required for persons with multiple risk factors or, in those with 0-1 risk factor, if the LDL-C level is only slightly below the goal level	High risk: CHD, or CHD risk equivalent including PAD, carotid artery disease, abdominal aortic aneurysm, type 2 diabetes, 10-y Framingham global risk of >20% due to multiple risk factors including cigarette smoking, hypertension, low HDL-C, family history of premature CHD, aged ≥55 y for women	Optimal/Desirable: TC <200 mg/dL, LDL-C <100 mg/dL, HDL-C ≥60 mg/dL, TG <150 mg/dL Above optimal: LDL-C = 100-129 mg/dL Borderline high: TC = 200-239 mg/dL, LDL-C = 130-159 mg/dL, TG = 150-199 mg/dL High: TC ≥240 mg/dL, LDL-C = 160-189 mg/dL, HDL-C <40 mg/dL, TG = 200-499 mg/dL Very high: LDL-C ≥190 mg/dL, TG ≥500 mg/dL
USPSTF (64) 2008	WHO: At-risk women aged 20-45 y WHEN: Healthy adults: no recommendation At-risk adults: uncertain; reasonable options include every 5 y, shorter intervals for people who have lipid levels close to those warranting therapy, and longer intervals for those not at increased risk with repeatedly normal lipid levels	Diabetes, previous personal history of CHD or noncoronary atherosclerosis, family history of CVD before age 50 in male relatives or age 60 in female relatives, tobacco use, hypertension, obesity	TC and HDL-C (fasting or nonfasting) Confirm abnormal screening test results with a repeated sample on a separate occasion, and the average of both results should be used for risk assessment
VHA (65) 2006	WHO: All adult women aged ≥45 y and adult women <45 y with ≥1 risk factors WHEN: Healthy adults: every 5 years At-risk adults: more often if family history of premature CVD exists	Older age, family history of premature CVD, hypertension, or under treatment for hypertension, smoking, diabetes mellitus, abdominal obesity	Fasting lipid profile including TC ≥240 mg/dL, HDL-C <40 mg/dL, TG >200 mg/dL, LDL-C ≥130 mg/dL, if calculated but consider direct measurement of LDL-C if TG >400 mg/dL

Abbreviations: NHLBI, National Heart, Lung, and Blood Institute; JNC 7, the Joint National Committee on Prevention, Detection, Evaluation, and Treatment of High Blood Pressure; AAFP, American Academy of Family Physicians; SBP, systolic blood pressure; DBP, diastolic blood pressure; CHD, coronary heart disease; PAD, peripheral artery disease; CVD, cardiovascular disease; PAD, peripherial artery disease; LDL-C, low-density lipoprotein cholesterol; HDL-C, high-density lipoprotein cholesterol; GFR, glomerular filtration rate; TG, triglycerides; TC, total cholesterol.

## References

[1] Heron M, Hoyert DL, Murphy SL, Xu J, Kochanek KD, Tejada-Vera B (2009). Deaths: final data for 2006.

[2] Health Data Interactive.

[3] Yoon SSS, Ostechega Y, Louis T (2010). Recent trends in the prevalence of high blood pressure and its treatment and control, 1999-2008. NCHS data brief.

[4] Duckitt K, Harrington D (2005). Risk factors for pre-eclampsia at antenatal booking: systematic review of controlled studies. BMJ.

[5] Ferrer RL, Sibai BM, Mulrow CD, Chiquette E, Stevens KR, Cornell J (2000). Management of mild chronic hypertension during pregnancy: a review. Obstet Gynecol.

[6] Livingston JC, Maxwell BD, Sibai BM (2003). Chronic hypertension in pregnancy. Minerva Ginecol.

[7] Simpson LL (2002). Maternal medical disease: risk of antepartum fetal death. Semin Perinatol.

[8] Phelan N, O'Connor A, Kyaw-Tun T, Correia N, Boran G, Roche HM (2010). Lipoprotein subclass patterns in women with polycystic ovary syndrome (PCOS) compared with equally insulin-resistant women without PCOS. J Clin Endocrinol Metab.

[9] Boomsma CM, Fauser BC, Macklon NS (2008). Pregnancy complications in women with polycystic ovary syndrome. Semin Reprod Med.

[10] Catov JM, Ness RB, Wellons MF, Jacobs DR, Roberts JM, Gunderson EP (2010). Prepregnancy lipids related to preterm birth risk: the Coronary Artery Risk Development in Young Adults Study. J Clin Endocrinol Metab.

[11] Bentley-Lewis R, Koruda K, Seely EW (2007). The metabolic syndrome in women. Nat Clin Pract Endocrinol Metab.

[12] Kelley GA, Kelley KS, Tran ZV (2004). Aerobic exercise and lipids and lipoproteins in women: a meta-analysis of randomized controlled trials. J Womens Health (Larchmt).

[13] Kelley GA, Kelley KS (1999). Aerobic exercise and resting blood pressure in women: a meta-analytic review of controlled clinical trials. J Womens Health Gend Based Med.

[14] Yu-Poth S, Zhao G, Etherton T, Naglak M, Jonnalagadda S, Kris-Etherton PM (1999). Effects of the National Cholesterol Education Program's Step I and Step II dietary intervention programs on cardiovascular disease risk factors: a meta-analysis. Am J Clin Nutr.

[15] Pergolini MS (2009). The management of hypertensive crises: a clinical review. Clin Ter.

[16] McPherson R, Genest J, Angus C, Murrary P (2001). The Women's Atorvastatin Trial on Cholesterol (WATCH): frequency of achieving NCEP-II target LDL-C levels in women with and without established CVD. Am Heart J.

[17] Pignone MP, Phillips CJ, Atkins D, Teutsch SM, Mulrow CD, Lohr KN (2001). Screening and treating adults for lipid disorders. Am J Prev Med.

[18] Cook NR, Cutler JA, Obarzanek E, Buring JE, Rexrode KM, Kumanyika SK (2007). Long term effects of dietary sodium reduction on cardiovascular disease outcomes: observational follow-up of the trials of hypertension prevention (TOHP). BMJ.

[19] Treadwell JR, Tregear SJ (2006). A system for rating the stability and strength of medical evidence. BMC Med Res Methodol.

[20] Gerhard GT, Connor SL, Wander RC, Connor WE (2000). Plasma lipid and lipoprotein responsiveness to dietary fat and cholesterol in premenopausal African American and white women. Am J Clin Nutr.

[21] Pellizzer AM, Straznicky NE, Lim S, Kamen PW, Krum H (1999). Reduced dietary fat intake increases parasympathetic activity in healthy premenopausal women. Clin Exp Pharmacol Physiol.

[22] Ginsberg HN, Kris-Etherton P, Dennis B, Elmer PJ, Ershow A, Lefevre M (1998). Effects of reducing dietary saturated fatty acids on plasma lipids and lipoproteins in healthy subjects: the DELTA Study, protocol 1. Arterioscler Thromb Vasc Biol.

[23] Ciolac EG, Bocchi EA, Bortolotto LA, Carvalho VO, Greve J, Guimarães GV (2010). Effects of high-intensity aerobic interval training vs moderate exercise on hemodynamic, metabolic and neuro-humoral abnormalities of young normotensive women at high familial risk for hypertension. Hypertens Res.

[24] Boreham CA, Kennedy RA, Murphy MH, Tully M, Wallace WF, Young I (2005). Training effects of short bouts of stair climbing on cardiorespiratory fitness, blood lipids, and homocysteine in sedentary young women. Br J Sports Med.

[25] Kin Isler, Kosar SN, Korkusuz F (2001). Effects of step aerobics and aerobic dancing on serum lipids and lipoproteins. J Sports Med Phys Fitness.

[26] LeMura LM, von Duvillard SP, Andreacci J, Klebez JM, Chelland SA, Russo J (2000). Lipid and lipoprotein profiles, cardiovascular fitness, body composition, and diet during and after resistance, aerobic and combination training in young women. Eur J Appl Physiol.

[27] Prabhakaran B, Dowling EA, Branch JD, Swain DP, Leutholtz BC (1999). Effect of 14 weeks of resistance training on lipid profile and body fat percentage in premenopausal women. Br J Sports Med. Br J Sports Med.

[28] Santiago MC, Leon AS, Serfass RC (1995). Failure of 40 weeks of brisk walking to alter blood lipids in normolipemic women. Can J Appl Physiol.

[29] Boyden TW, Pamenter RW, Going SB, Lohman TG, Hall MC, Houtkooper LB (1993). Resistance exercise training is associated with decreases in serum low-density lipoprotein cholesterol levels in premenopausal women. Arch Intern Med.

[30] Hinkleman LL, Nieman DC (1993). The effects of a walking program on body composition and serum lipids and lipoproteins in overweight women. J Sports Med Phys Fitness.

[31] Duncan JJ, Gordon NF, Scott CB (1991). Women walking for health and fitness. How much is enough?. JAMA.

[32] Edin JB, Gerberich SG, Leon AS, McNally C, Serfass R, Shaw G (1990). Analysis of the training effects of minitrampoline rebounding on physical fitness, body composition, and blood lipids. J Cardiopulm Rehabil Prev.

[33] Duey WJ, O'Brien WL, Crutchfield AB, Brown LA, Williford HN, Sharff-Olson M (1998). Effects of exercise training on aerobic fitness in African-American females. Ethn Dis.

[34] Katz J, Wilson BR (1992). The effects of a six-week, low-intensity Nautilus circuit training program on resting blood pressure in females. J Sports Med Phys Fitness.

[35] Oluseye KA (1990). Cardiovascular responses to exercise in Nigerian women. J Hum Hypertens.

[36] Janssen I, Fortier A, Hudson R, Ross R (2002). Effects of an energy-restrictive diet with or without exercise on abdominal fat, intermuscular fat, and metabolic risk factors in obese women. Diabetes Care.

[37] Ågren JJ, Pekkarinen H, Litmanen H, Hänninen O (1991). Fish diet and physical fitness in relation to membrane and serum lipids, prostanoid metabolism and platelet aggregation in female students. Eur J Appl Physiol Occup Physiol.

[38] Wood PD, Stefanick ML, Williams PT, Haskell WL (1991). The effects on plasma lipoproteins of a prudent weight-reducing diet, with or without exercise, in overweight men and women. N Engl J Med.

[39] Esposito K, Pontillo A, Di Palo C, Giugliano G, Masella M, Marfella R, Giugliano D (2003). Effect of weight loss and lifestyle changes on vascular inflammatory markers in obese women: a randomized trial. JAMA.

[40] Fogelholm M, Kukkonen-Harjula K, Nenonen A, Pasanen M (2000). Effects of walking training on weight maintenance after a very-low-energy diet in premenopausal obese women: a randomized controlled trial. Arch Intern Med.

[41] Chobanian AV, Bakris GL, Black HR, Cushman WC, Green LA, Izzo JL (2003). Seventh report of the Joint National Committee on Prevention, Detection, Evaluation, and Treatment of High Blood Pressure. Hypertension.

[42] American Academy of Family Physicians. Summary of recommendations for clinical preventive services, 2011.

[43] Mosca L, Benjamin EJ, Berra K, Bezanson JL, Dolor RJ, Lloyd-Jones DM (2011). Effectiveness-based guidelines for the prevention of cardiovascular disease in women — 2011 update: a guideline from the American Heart Association. Circulation.

[44] Institute for Clinical Systems Improvement. Hypertension diagnosis and treatment. 2008 October. Report No: Twelfth Edition.

[45] Institute for Clinical Systems Improvement. Preventive services for adults. 2009 October. Report No.: Fifteenth Edition..

[46] US Preventive Services Task Force (2007). Screening for high blood pressure: U.S. Preventive Services Task Force reaffirmation recommendation statemen. Ann Intern Med.

[47] Department of Veterans Administration, Department of Defense. VA/DoD clinical practice guideline for the diagnosis and management of hypertension in the primary care setting.

[48] National Cholesterol Education Program, National Heart, Lung, and Blood Institute, National Institutes of Health (2002). Third Report of the National Cholesterol Education Program (NCEP) Expert Panel on Detection, Evaluation, and Treatment of High Blood Cholesterol in Adults (Adult Treatment Panel III) 2002. Circulation.

[49] The Emerging, Di Angelantonio, Sarwar N, Perry P, Kaptoge S, Ray KK (2009). Major lipids, apoloipoproteins, and risk of vascular disease. JAMA.

[50] Wilcox LS, Mosher WD (1993). Factors associated with obtaining health screening among women of reproductive age. Public Health Rep.

[51] Kuklina EV, Yoon PW, Keenan NL (2010). Prevalence of coronary heart disease risk factors and screening for high cholesterol levels among young adults, United States, 1999-2006. Ann Fam Med.

[52] Fleming P, Godwin M (2008). Lifestyle interventions in primary care: systematic review of randomized controlled trials. Can Fam Physician.

[53] Kelley GA (1999). Aerobic exercise and resting blood pressure among women: a meta-analysis. Prev Med.

[54] Yoshida H, Ikshikawa T, Suto M, Kurosawa H, Hirowatari Y, Ito K (2010). Effects of supervised aerobic exercise training on serum adiponectin and parameters of lipid and glucose metabolism in subjects with moderate dyslipidemia. J Atheroscler Thromb.

[55] Kodama S, Tanaka S, Saito K, Shu M, Sone Y, Onitake F (2007). Effect of aerobic exercise training on serum levels of high-density lipoprotein cholesterol: a meta-analysis. Arch Intern Med.

[56] Natarajan P, Ray KK, Cannon CP (2010). High-density lipoprotein and coronary heart disease: current and future therapies. J Am Coll Cardiol.

[57] Hamer M, Chida Y (2008). Walking and primary prevention: a meta-analysis of prospective cohort studies. Br J Sports Med.

[58] Oguma Y, Shinoda-Tagawa T (2004). Physical activity decreases cardiovascular disease risk in women: review and meta-analysis. Am J Prev Med.

[59] Sofi F, Capalbo A, Cesari F, Abbate R, Gensini GF (2008). Physical activity during leisure time and primary prevention of coronary heart disease: an updated meta-analysis of cohort studies. Eur J Cardiovasc Prev Rehabil.

[60] Centers for Disease Control and Prevention (2005). Disparities in screening for and awareness of high blood cholesterol — United States, 1999-2002. MMWR Morb Mortal Wkly Rep.

[61] Nelson K, Norris K, Mangione CM (2002). Disparities in the diagnosis and pharmacologic treatment of high serum cholesterol by race and ethnicity: data from the Third National Health and Nutrition Examination Survey. Arch Intern Med.

[62] (2007). Guidelines for women's health care: a resource manual.

[63] Lipid management in adults. 2009. Report No: Eleventh edition. Institute for Clinical Systems Improvement.

[64] (2008). Screening for lipid disorders in adults.

[65] VA/DoD clinical practice guideline for the management of dyslipidemia; 2006.

